# Supplementary material for: A Hypothesis of Gut–Liver Mediated Heterosis: Multi-Omics Insights into Hybrid Taimen Immunometabolism (Hucho taimen ♀ × Brachymystax lenok ♂)
Source: Animals (Basel). 2025 Dec 26;16(1):74. doi: 10.3390/ani16010074 (PMC12785115; doi:10.3390/ani16010074)
Supplement: Supplementary file 1 [file animals-16-00074-s001.zip › animals-4042367-supplementary.pdf]

1.

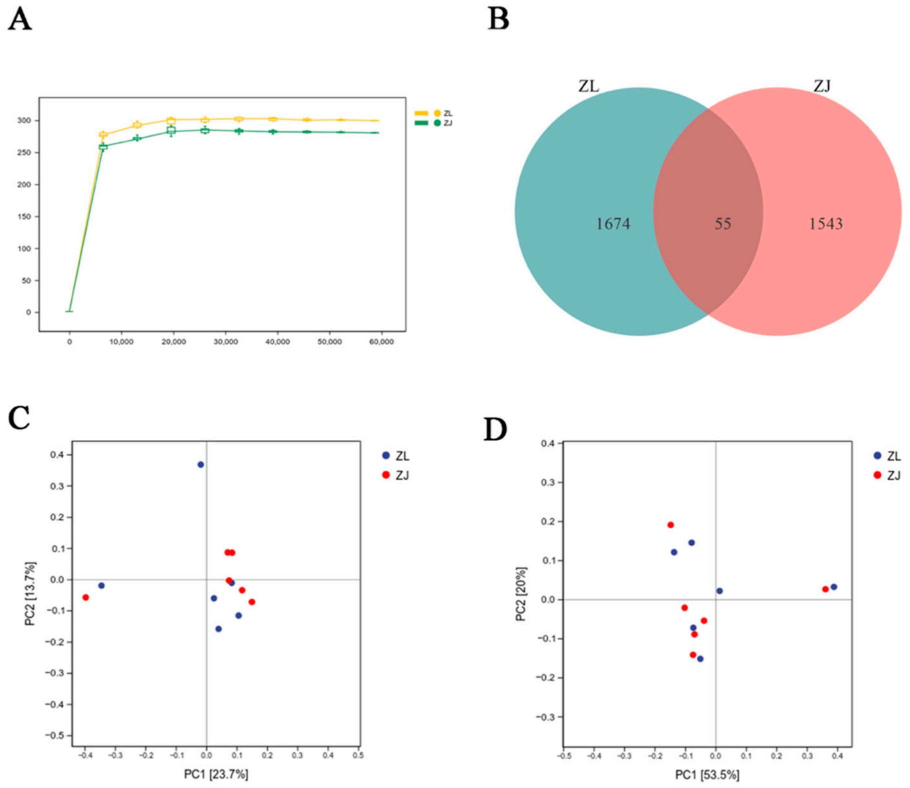

Figure S1. Richness and diversity of intestinal microbial communities in *Hucho taimen* (ZL) and hybrid taimen (ZJ). (A) Rarefaction curve analysis of intestinal microbiota. (B) Venn diagram showing the number of shared and unique OTUs in the intestinal microbiota of Hucho taimen and hybrid Hucho taimen. (C) β-diversity indicated by PCoA based on unweighted UniFrac distance and (D) weighted UniFrac distance.

2. Table S1: Top 10 microbial taxa by abundance at the phylum level in the intestines of *Hucho taimen* (ZL) and hybrid taimen (ZJ).

Taxonomy	ZL	ZJ
p_Bacillota	0.47468650	0.48311750
p_Pseudomonadota	0.36684933	0.36808950
p_Cyanobacteriota	0.07251683	0.07821050
p_Actinomycetota	0.06573233	0.04689983
p_Mycoplasmatota	0.00909950	0.01179917
p_Planctomycetota	0.00436650	0.00535000
p_Bacteroidota	0.005916170	0.00343317
p_Campylobacterota	0.000483330	0.00305000

p_Thermodesulfobacteriota	0.000333330	0.00003330
p_Myxococcota	0.000000000	0.00001670
p_Acidobacteriota	0.000016700	0.000000000
Others	0.000000000	0.000000000

3. Table S2: Top 20 microbial taxa by abundance at the genus level in the intestines of *Hucho taimen* (ZL) and hybrid taimen (ZJ).

Taxon	ZL	ZJ
g_Marinobacterium	0.08144817	0.15753050
g_Enterococcus	0.05739033	0.09770850
g_Streptococcus	0.07117200	0.03262317
g_Bacillus	0.03360383	0.04097500
g_Paracoccus	0.04678683	0.02337133
g_Pseudomonas	0.02734000	0.03650767
g_Staphylococcus	0.04539667	0.01830417
g_Clostridium	0.04370600	0.00991900
g_Tepidimicrobium	0.00892900	0.04070950
g_Hapalosiphon	0.00538300	0.04047067
g_Sulfitobacter	0.02036567	0.02503833
g_Vagococcus	0.01969850	0.02322233
g_Shewanella	0.02999783	0.01290300
g_Toxopsis	0.03427983	0.00498483
g_Anaerococcus	0.01741133	0.02090617
g_Sanguibacter	0.00924967	0.02757067
g_Tepidibacter	0.01930083	0.01658817
g_Aerococcus	0.02012933	0.01492167
g_Synechococcus	0.02536233	0.00701850
g_Aeromonas	0.02273433	0.00845250
Others	0.36031450	0.34027433

4. Table S3 RNA-Seq sequencing results of intestine transcriptome.

Sample	Raw Reads	Clean Data(bp)	Clean_Reads	GC(%)	N(%)	Q20(%)	Q30(%)	Mapped reads	Mapped rate(%)
ZLC1	38648318	5700381530	37901026	46.89	0.009994	98.67	95.05	33031865	87.15%
ZLC2	48346056	7081995676	47075212	46.97	0.010284	98.38	94.09	40141995	85.27%
ZLC3	38867348	5745885631	38180106	46.56	0.012694	98.79	95.56	32988161	86.40%
ZLG1	38498132	5705650835	37900054	48.18	0.012763	98.91	95.75	33212988	87.63%
ZLG2	44514850	6579546725	43838548	47.73	0.012699	98.88	95.73	37488122	85.51%
ZLG3	42630338	6329790450	42065054	48.22	0.030936	98.94	96.08	35212816	83.71%
ZJC1	37808910	5588790897	37135908	46.85	0.012646	98.80	95.62	29080864	78.31%
ZJC2	35841972	5308597446	35264422	46.62	0.012683	98.85	95.63	27546951	78.12%
ZJC3	41071680	6079752796	40393040	46.57	0.012670	98.82	95.58	31322256	77.54%

ZJG1	51616254	7643852677	50764774	48.38	0.012577	98.86	95.65	40475650	79.73%
ZJG2	47840834	7095814396	47117548	48.54	0.010322	98.86	95.53	37223349	79.00%
ZJG3	77433520	11490689850	76328004	48.14	0.007261	98.82	95.34	59884752	78.46%

5. Table S4 qRT-PCR Primer Information

Number	Primer Name	Forward primer 5'-3'	Right primer 5'-3'	Length of production/bp
1	*smpd1*	AGGCCAGTGAAGAGAAGGG	ATAGGTGGTGACGGAGGGA	233
2	*gstp1*	ATGGAAAAGATGGCAAGGA	TAGTCAGCAAACGAAGGCT	217
3	*f9a*	TATGCTGAGCACTTGTTGG	TGTGTAGGGGCTATTTGAC	153
4	*bmpr1bb*	TAACCCCTTCAAAACCCGT	GACTTCCCTTCCAACAGCA	213
5	*Aldh6a1*	AAGTGTTGGTATTTGTGAG	AGAAAGTGTGTTTGGAGAG	237
6	*tcirg1b*	GAGGCAGGGAGTCACAGAG	CGAAGGCGAAGAAAAATAG	247
7	*Hyal2*	TCTTTCTCAGTCTCCTTCC	AACTATTTTGTGCTATTCC	207
8	*cyp1a*	GCCGTTCACCATCCCACAC	TACGAGCACCTTCTCCCCC	205
9	*fmoda*	ACATTATCATACATCTCCA	ACAACAGTTACATCCCTAC	247
10	*aldh3a1*	GTCGCATCATCAACCTACG	GCATCACCCACATCACTAA	205
11	*fdft1*	CACTCAACCCCCACTAACC	AAACACAAGACAACCCTGC	191
12	*hadhab*	GGACTCAAAAGCAAAGACG	GAATCCCAGACCAAACACA	207
13	*rxraa*	CACAAAATGAAGAAGAAAT	AGAAGAGTGGAAACAAAAG	151
14	*gstt1b*	TATTGTCCCTCTGTGCCCT	CCTGACCCACCTGTATCCC	163
15	*npc1l1*	AGGGTCATAGTGATGGTTG	CATTGTGCTGGTGAAGTTA	207
16	*lbr*	CTAACAAAGTCAACGAGAA	AGCAGAGTCATACAGAACA	211
17	*gcdha*	ACCCATCTATCTGCCTTCT	ACCATTGTGACATCCCTTC	241
18	*pltp*	AACCCTTTCAGTTTCTCAC	TCTTTACGGTATCTCCACA	183
19	*ugt5g1*	TCAATAAAAACTGACAAAA	TGACATCATAGAGAGGAGA	151
20	*scarb1*	GTTGTGGAAAACATAGGGT	TGGTGTAGTGAGCAGTCAG	179
21	*β-Actin*	TCTACGAAGGCTACGCTCTG	CAGCTTCTCCTTGATGTCGC	155
